# The innate immune system: A double-edged sword

**DOI:** 10.1371/journal.pbio.3003088

**Published:** 2025-04-29

**Authors:** Ditte S. Andersen, Julien Colombani

**Affiliations:** Department of Biology, University of Copenhagen, Copenhagen, Denmark

## Abstract

Innate immunity serves as a crucial surveillance framework, but can be exploited to facilitate tumor progression. Two new PLOS Biology studies independently show how premalignant cells can exploit Toll-NF-κB signaling, in concert with oncogenic Ras, to enable unchecked growth

Innate immunity serves as a global surveillance system, sensing external stimuli and assessing cellular fitness to initiate context-specific responses that combat infections and maintain tissue homeostasis. The Toll pathway, conserved from invertebrates to mammals, is a crucial component of the innate immune system. Its activation triggers a signaling cascade that leads to the translocation of the transcription factor, NF-κB (Dorsal and Dif in *Drosophila*), into the nucleus, driving the production of host defense peptides. Beyond its role in immunity, the Toll pathway has also been linked to cell competition - a process that eliminates less fit cells through apoptosis to uphold tissue homeostasis [1,2]. Notably, the proinflammatory properties of innate immune signals can be exploited by malignant cells to facilitate tumor progression. In Ras^v12^/scrib^-*/-*^ tumors, this is exemplified by their ability to hijack Tumor Necrosis Factor (TNF)/Eiger to enhance tumor growth and invasion [3,4]. The Ras^V12^*/scrib*^*−/−*^ tumor model has been widely utilized in *Drosophila* cancer research, as it exhibits a striking array of hallmarks reminiscent of human malignancies [5]. In this model, the combination of oncogenic Ras (Ras^V1*2*^) expression with loss of cell polarity due to *scribble* loss-of-function results in malignant cells that are refractory to apoptosis, leading to the formation of aggressive, invasive tumors (Fig 1) [6,7]. Similarly, Toll-like receptor (TLR) signaling is frequently hyperactivated in malignant cells [8], although the mechanisms by which Toll pathway synergizes with cancer-driving mutations to promote tumor growth remain unclear. To explore this, two recent studies in *PLOS Biology*, Brutscher F. and colleagues and Dillard C. and colleagues, employed *Drosophila* Ras^V12^-driven epithelial tumor models to investigate how Toll-NF-κB signaling cooperates with oncogenic Ras to drive unrestricted growth and invasiveness [9,10].

**Fig 1:**
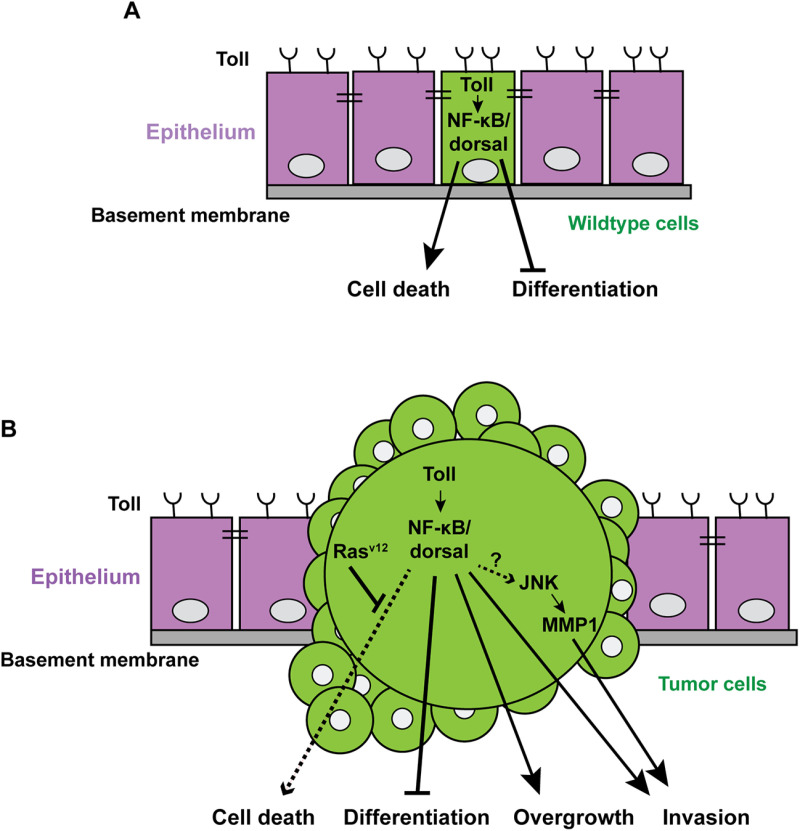
The Toll-NF-κB pathway synergizes with oncogenic lesions to promote tumorigenesis. (**A**) While Toll-NF-κB pathway activation alone inhibits differentiation, trapping cells in a progenitor-like state, it also increases cell death rates leading to an overall reduction in tissue size. (**B**) In the presence of oncogenic Ras^V12^, these progenitor-like cells become refractory to cell death, which drives tumor growth. Additionally, NF-κB/Dorsal activates JNK signaling and enhances the invasiveness of polarity-deficient Ras^V12^/*scrib*^*−/−*^ tumors.

Brutscher and colleagues show that while Toll-NF-κB pathway activation alone reduces tissue size, its activation in conjunction with oncogenic Ras^V12^ creates a potent oncogenic synergy, driving tissue overgrowth. Using RNA sequencing, they identified genes that are differentially expressed upon co-expression of Ras^V12^ and Dorsal (Dl) [10]. Their analyses reveal that Toll pathway activation suppresses differentiation markers; a finding they experimentally validate in vivo. Additionally, they show that although Toll-NF-κB signalling induces apoptosis, its activation alongside with Ras^V12^, which inhibits apoptosis, shifts the effect toward increased cell proliferation. Notably, despite upregulation of several JNK/AP-1 target genes, JNK is not required downstream of Dorsal to amplify Ras^V12^-driven tissue overgrowth.

Through RNA sequencing of Ras^v12^/*scrib*^*−/−*^ tumors, Dillard and colleagues identify several Toll pathway components as upregulated and demonstrate an autonomous requirement for Toll-NF-κB signaling to promote Ras^v12^/*scrib*^*−/−*^ tumor growth. Focusing on Dl, the authors examine the two alternatively spliced isoforms, DlA and DlB, both of which are upregulated in Ras^v12^/*scrib*^*−/−*^ tumors. Notably, these isoforms display an asymmetric distribution, with elevated expression in the posterior region of the tumor near the central nervous system and the highest levels in tumor cells actively invading neighbouring tissues. Furthermore, DlA and DlB display a mutually exclusive expression pattern with DlB being expressed in neurons within the tumor and DlB in non-neuronal cells surrounding these. Intriguingly, DlB expression shows a positive correlation with JNK activity, a key driver of Ras^v12^/*scrib*^*−/−*^ tumor invasiveness. Consistent with this, they show that silencing of Dl partially restores retinal differentiation, increases apoptosis and reduces tumor invasion. These findings raise the intriguing possibility that each isoform could serve a distinct function, with DlB driving JNK-mediated invasion and DlA supporting stemness and survival. Uncovering the mechanisms that regulate differential Dl splicing and how this contributes to tumorigenesis presents an interesting line of research.

Altogether, Brutscher F. and colleagues and Dillard C. and colleagues demonstrate that the Toll-NF-κB signaling cooperates with cancer-driving mutations to suppress differentiation, counteract apoptosis, and promote invasion [9,10]. One open question remains regarding how the innate immune system is activated in these tumors. Intriguingly, results from a recent study using a Yorkie-driven intestinal tumor model suggest that innate immunity may be triggered through interactions between the tumor and its microenvironment. Their findings shows that TNF/Eiger production from Yki^act^ tumors induces cell-competition-driven apoptosis in neighboring non-tumor cells. This, in turn, initiates an NF-κB-driven inflammatory response within the tumor, possibly mediated by damage-associated molecular patterns released from dying cells [11].

The role of the Toll-NF-κB signalling pathway in tumor growth parallels that of another innate immune signal, TNF, which can function as both a tumor promoter and suppressor. In *Drosophila*, the TNF homolog, Eiger, plays a key role in an antitumor surveillance system that facilitates the elimination of premalignant cells from peripheral tissues. However, like Toll-NF- κB signalling, Eiger/TNF can also be repurposed into a protumor signal with harmful consequences for the host. For instance, the invasive behaviour of Ras^V12^*/scrib*^*−/−*^ tumors is driven by Eiger/TNF, which activates JNK signalling and drives the expression of matrix metalloproteinase-1, promoting tumor invasion and progression [3,4].

The observed heterogeneity of NF-kB/Dl levels and activity mirrors patterns observed in mammalian cancers and raises important questions regarding the differential immune response of malignant cells. Understanding whether this heterogeneity stems from extrinsic signals from nearby cells in the microenvironment or intrinsic regulatory mechanisms, as well as how intratumoral NF-kB heterogeneity contributes to malignant transformation, presents an exciting avenue for future research.
